# Sex-Based Variations in Metal(loid) Levels in Green Tiger Shrimp (*Penaeus semisulcatus*, Decapoda:Penaeidae) from the Northeastern Mediterranean Coast of Türkiye: A Human Health Risk-Benefit Assessment

**DOI:** 10.3390/life16030487

**Published:** 2026-03-17

**Authors:** Mustafa Gocer, Mine Percin Olgunoglu, Ilkan Ali Olgunoglu

**Affiliations:** Kahta Vocational Training School, Veterinary Department, Adiyaman University, Adiyaman 02040, Türkiye; mgocer@adiyaman.edu.tr (M.G.); iolgunoglu@adiyaman.edu.tr (I.A.O.)

**Keywords:** *Penaeus semisulcatus*, heavy metals, health risk assessment, shrimp, Mediterranean

## Abstract

This study provides a comprehensive assessment of 12 metal(loid)s in the muscle tissue of the commercially vital shrimp, *Penaeus semisulcatus*, from four stations (Bozyazi, Silifke, Karatas, and Iskenderun) along the Northeastern Mediterranean. Metal concentrations were evaluated separately for males and females, utilizing Estimated Weekly Intake (EWI), Target Hazard Quotient (THQ), Carcinogenic Risk (CR), and Selenium Health Benefit Value (HBV_Se_) indices. While the species is generally safe for consumption across the region, a striking, localized bioaccumulation of Chromium (Cr) was identified specifically in Iskenderun Bay, where male shrimps exhibited concentrations (1.209 mg/kg wet weight) approximately 10-fold higher than females, highlighting a sex-specific sensitivity likely linked to metabolic and physiological differences. By adopting a precautionary risk assessment framework—considering the region’s intense industrial profile—this localized spike resulted in a Total Carcinogenic Risk (∑CR = 5.15 × 10^−4^) for this group, exceeding the priority threshold. Furthermore, widespread Lead (Pb) contamination was detected across all stations, with several samples surpassing EU maximum levels (0.50 mg/kg). Regarding Arsenic (As), while high total concentrations led to THQ values > 1 across the regional gradient, this was characterized as a conservative modeling artifact rather than a physiological threat, as Arsenic in crustaceans is predominantly in the non-toxic organic form. Conversely, any potential risk from Mercury (Hg) was conclusively mitigated by an overwhelming molar excess of Selenium (Se) at all locations, confirmed by consistently positive HBV_Se_ values (0.312–0.658). In conclusion, our findings demonstrate that seafood safety is conditional and region-specific. The study underscores that localized contamination “hotspots” can be easily masked by non-sex-specific sampling and emphasizes the necessity of moving beyond simplistic risk models by incorporating selenium-mercury antagonism and precautionary risk assumptions for industrial pollutants.

## 1. Introduction

Marine ecosystems are vital yet fragile environments that are increasingly endangered by anthropogenic influences, particularly in regions affected by industrial activities and the unregulated discharge of pollutants [1,2]. Contaminants, both organic and inorganic, disrupt the ecological balance of aquatic environments [1]. In recent decades, there has been growing concern regarding the presence of potentially toxic trace elements (TEs) in the marine environment and the health implications for humans consuming seafood with elevated TE levels [3]. Due to their persistence and bio accumulative nature, TEs can remain in the environment for prolonged periods and accumulate in humans via the food chain, potentially posing serious hazards. Chronic exposure to these elements has been linked to severe health issues, including renal toxicity, hepatic damage, as well as neurological and cardiovascular diseases [1].

Conversely, seafood is globally recommended as a key nutritional source, rich in proteins, long-chain omega-3 polyunsaturated fatty acids (PUFAs), and essential minerals [2,3]. However, this nutritional benefit creates a “toxicological dilemma” due to the simultaneous presence of heavy metals. In this context, numerous studies have focused on Human Health Risk-Benefit Assessments to balance the nutritional advantages against the risks of heavy metal contamination in various aquatic products [4,5,6,7,8,9,10,11,12,13,14,15,16,17,18]. Among marine organisms, crustaceans—particularly shrimps—are widely utilized as biological indicators to assess coastal water quality and the biological effects of contaminants [19,20]. Unlike many fish species, shrimps are scavengers that consume detritus and benthic materials, a feeding habit that increases their exposure to sediment-bound trace elements [20,21]. Their benthic lifestyle and relatively limited migration patterns make them excellent indicators of localized, site-specific environmental contamination, as their tissue metal concentrations can directly reflect the pollution status of their immediate habitat [3].

*Penaeus semisulcatus* (green tiger shrimp) is a commercially important species prevalent along the Eastern Mediterranean coast of Türkiye [21,22]. This coastal region is characterized by intensive anthropogenic activities and is exposed to significant pollution loads from industrial facilities, petroleum transfer harbors, and agricultural runoff, particularly in industrial hotspots such as Iskenderun Bay and surrounding areas [2,3]. Given this regional pollution pressure, determining the accumulation levels of TEs in *P. semisulcatus* is critical for assessing food safety risks.

The accumulation of trace elements in marine species is governed by a complex interplay of biological factors, including feeding habits, trophic position, age, and metabolic requirements [1,19]. Notably, recent evidence suggests that variability in metal concentrations may be significantly influenced by the sex of the individual [21,23]. Sex is a key biotic factor that modulates metal bioaccumulation and tissue distribution, largely due to differences in metabolic rates, hormonal profiles, and energy expenditure during reproductive cycles [21]. Prior research on *P. semisulcatus* has indicated that metal concentrations can vary significantly based on tissue type, season, and sex [21,24]. Furthermore, conflicting reports regarding metal levels in the same species from the same region underscore a persistent gap in the literature [25]. Elucidating the influence of sex is therefore essential for increasing the accuracy of human health risk assessments and ensuring they are not based on overgeneralized data.

The primary objective of this study is to compare the concentrations of heavy metals and metalloids in the edible tissues of female and male *P. semisulcatus* individuals collected from four distinct locations (Bozyazi, Silifke, Karatas, and Iskenderun) along the Northeastern Mediterranean coast of Türkiye. To achieve this aim, the following specific tasks were undertaken: (1) to quantify and compare the levels of 12 metal(loid)s in muscle tissue based on sex and sampling station; (2) to identify potential sex-specific bioaccumulation patterns; and (3) to conduct a comprehensive human health risk-benefit analysis based on these sex-differentiated data, incorporating the crucial interaction between selenium and mercury.

## 2. Materials and Methods

### 2.1. Study Area and Sampling Strategy

**Figure 1:**
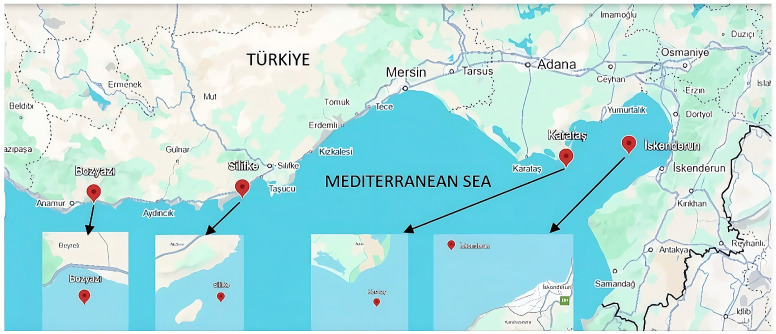
Sampling locations off the coasts of Bozyazi (36°04′49” N 32°57′38” E), Silifke (36°11′56″ N 33°47′58″ E), Karatas (36°31′39″ N 35°37′25″ E), and Iskenderun (36°37′14″ N 35°58′42″ E) in the Northeastern Mediterranean, Türkiye.

The study was conducted along the Northeastern Mediterranean coast of Türkiye, selecting four distinct stations (Bozyazi, Silifke, Karatas, and Iskenderun) to represent varying degrees of environmental and anthropogenic pressures. The geographical coordinates and the spatial distribution of these sampling stations are detailed in Figure 1. Among the selected sites, Bozyazi (Station 1) was considered a reference station, representing an area with relatively lower direct industrial and anthropogenic pressures compared to the other locations, particularly the heavily industrialized Iskenderun Bay (Station 4). 

Sampling was carried out during November and December of 2025. Sampling was timed to coincide with the post-spawning period (November–December) to minimize physiological variability associated with reproductive gonadal development, and care was taken to select inter-molt specimens to avoid variations in metal accumulation caused by the ecdysis (molting) process. A total of 120 specimens of Green Tiger Shrimp (*P. semisulcatus*) were collected using commercial trawling nets. To ensure statistical robustness and compare biological variables, approximately 30 individuals were collected from each station, with an equal sex distribution (15 males and 15 females per location).

Immediately after collection, the samples were rinsed with ambient seawater to remove extraneous matter and debris. The specimens were then placed in labeled polyethylene bags and transported to the laboratory in isothermal containers maintained at 4 °C using crushed ice. Upon arrival, individual shrimps were identified to the species level. Total length (TL) was measured from the tip of the rostrum to the end of the telson to the nearest 0.1 mm using a digital caliper, and whole weight (WW) was recorded to the nearest 0.01 g using an analytical balance. Sex was determined visually by identifying the presence of the petasma (male reproductive organ) in males and the thelycum (female reproductive organ) in females. The edible abdominal muscle tissues were carefully dissected from each specimen. To obtain representative baseline data for each group, the muscle tissues of the 15 individuals of the same sex from a given station were pooled (mixed) together and homogenized. This procedure resulted in a total of 8 composite samples (4 stations × 2 sexes). These composite homogenates were stored at −20 °C until chemical analysis.

### 2.2. Microwave Digestion Procedure

Sample preparation was performed by acid decomposition of the tissue matrix using a microwave digestion system (Berghof MSW-4, Eningen, Germany). From each of the 8 composite homogenized samples, approximately 250 mg (wet weight) of the tissue was precisely weighed and transferred into digestion vessels. A mixture of 5 mL of 65% HNO_3_ and 1 mL of 37% HCl was added to each vessel (the addition of HCl ensured the stabilization of elements such as Hg). The vessels were left open for 10–15 min to allow for initial degassing and to prevent sudden pressure build-up.

The digestion process was carried out according to a specific multi-step thermal program:

1. Step 1: The temperature was increased to 160 °C over 5 min (ramp) and maintained at 160 °C for 5 min at 80% power and a maximum pressure of 40 bar.
2. Step 2: The temperature was then raised to 190 °C over 5 min (ramp) and held for 15 min at 90% power and 40 bar pressure.
3. Step 3: The process concluded with a controlled cooling phase to 50 °C.

Following digestion, the resulting solutions were diluted to a final volume of 10 mL using ultra-pure water. Procedural blanks, containing only the acid mixture, were prepared simultaneously using the same protocol to monitor for potential contamination.

### 2.3. Elemental Analysis

Elemental concentrations were quantified using an Inductively Coupled Plasma Mass Spectrometry (ICP-MS) instrument (Perkin Elmer NexION 350X, Waltham, MA, USA). The instrument was equipped with a Meinhard concentric nebulizer, a glass cyclonic spray chamber, and a nickel triple cone interface. The operational parameters were optimized as follows: RF power of 1500 W, plasma gas flow of 18.0 L/min, auxiliary gas flow of 1.2 L/min, and nebulizer gas flow of 0.68 L/min. The sample uptake rate was maintained at 1 mL/min.

The system operated in both Standard (STD) and Kinetic Energy Discrimination (KED) modes, utilizing helium (He) as a collision gas to minimize polyatomic interferences and ensure reliable multi-element determination. For analysis, aliquots from the 10 mL digested solutions were further diluted with ultra-pure water as required to comply with the instrument’s linear calibration range.

To ensure the reliability and precision of the analytical results, strict quality assurance and quality control (QA/QC) procedures were implemented. All reagents used were of analytical grade (Merck, Darmstadt, Germany). Calibration curves were constructed using multi-element standard solutions, achieving correlation coefficients (R^2^) greater than 0.999 for all analyzed elements. The accuracy of the method was validated by analyzing Certified Reference Material (NIST 2976, mussel tissue), with recovery rates found within acceptable limits. All samples were analyzed in triplicate to verify reproducibility. The limits of detection (LOD) and quantification (LOQ) were calculated as three and ten times the standard deviation of the blank measurements, respectively.

### 2.4. Human Health Risk Assessment

The potential health risks associated with the consumption of *P. semisulcatus* were evaluated for the adult population. The following parameters and equations were utilized:

#### 2.4.1. Estimated Daily and Weekly Intake (EDI & EWI)

To evaluate the potential non-carcinogenic health hazards associated with the consumption of shrimp, the Estimated Daily Intake (EDI) of the analyzed metal(loid)s was calculated using Equation (1), following the methodology described by Sadeghi et al. [26].

EDI = (MC × FDC)/BW(1)

where

MC is the mean concentration of the specific element in the shrimp muscle (mg/kg wet weight).

FDC represents the average daily consumption rate of shrimp (0.041 kg/day), as reported by Abd-Elghany et al. [27].

BW is the average body weight for an adult, taken as 70 kg in this study.

Subsequently, the Estimated Weekly Intake (EWI) was determined using Equation (2), as established by Alipour et al. [28], to facilitate a direct comparison with international regulatory benchmarks, such as the Provisional Tolerable Weekly Intake (PTWI):

EWI = EDI × 7(2)

(Note: EWI is expressed in mg/kg body weight/week)

#### 2.4.2. Non-Carcinogenic Health Risk Assessment (THQ and ∑THQ) and Target Carcinogenic Risk (CR)

To assess the potential non-carcinogenic health risks associated with the consumption of the analyzed samples, the Target Hazard Quotient (THQ) was calculated for individual elements using Equation (3), as described by Sadeghi et al. [26].

THQ = (EF × ED × FIR × C)/(RfDs × BW × ATn) × 10^−3^ (3)

Furthermore, the cumulative non-carcinogenic risk resulting from the simultaneous exposure to multiple contaminants was evaluated using the Total Target Hazard Quotient (∑THQ), calculated via Equation (4) [29].

∑THQ = THQ (As) + … + THQ (Zn)(4)

A THQ or ∑THQ value of <1 indicates that no adverse health effects are anticipated. Conversely, a value > 1 suggests that chronic exposure may pose a potential non-carcinogenic health risk, requiring further monitoring [30,31]. All exposure parameters and reference doses (RfD) used in these calculations are summarized in Table 1.

The lifetime cancer risk (CR) was estimated for elements classified as potential carcinogens by the International Agency for Research on Cancer (IARC), specifically As, Cd, Cr, Ni, and Pb. Considering that inorganic arsenic (As) is the primary toxic form in seafood, a conversion factor of 10% was applied to the total arsenic concentrations for the CR calculations, as suggested by Zhong et al. [32].

The CR was quantified using Equation (5) [33,34].

CR = (EF × ED × FIR × C × CSF)/(BW × ATc) × 10^−3^ (5)

where CSF represents the Cancer Slope Factor. The CSF values used for As, Cd, Cr, Ni, and Pb were 1.5, 0.01, 0.5, 1.7, and 0.38 mg kg^−1^ day^−1^, respectively. The acceptable threshold for individual carcinogenic risk is established as 10^−6^, while the cumulative limit for exposure to multiple carcinogenic elements is considered to be 10^−4^ [35].

**Table 1:** Parameters and values used for THQ and CR calculations.

Parameter	Symbol	Unit	Value (Adult)	Reference
Exposure Frequency	EF	days/year	365	-
Exposure Duration	ED	years	26	[36]
Food Ingestion Rate	FIR	g/day	41.0	[27]
Metal Concentration	C	mg/kg (Wet weight)	Present study	-
Average Body Weight	BW	kg	70	[37]
Averaging Time (Non-carcinogens)	ATn	days	9490	EF × ED
Averaging Time (Carcinogens)	ATc	days	25,550	70 years (life time × 365 days/year)
Oral Reference Dose	RfD	mg kg^−1^ day^−1^	* See below	[16,29,37,38]
Note: * Rfd Values: Pb = 0.0035; Ni = 0.02; Cd = 0.001; Cr = 0.003; Cu = 0.04; Fe = 0.7; Mn = 0.14; Zn = 0.3; Hg = 0.0001; Se = 0.005; As = 0.0003				

#### 2.4.3. Selenium (Se): Mercury (Hg) Molar Ratios and Se Health Benefit Value (HBV_Se_)

For a comprehensive assessment of seafood safety, the interaction between Se and Hg was evaluated using two established indices: the Se:Hg molar ratio and the Selenium Health Benefit Value (HBV_Se_). This approach is based on the widely accepted premise that Se exerts protective effects against Hg toxicity, providing a more robust basis for consumer risk analysis than total concentration measurements alone.

First, molar concentrations (μmol/kg) were derived by dividing the measured concentration (mg/kg) of each element by its respective atomic weight (Se: 78.96 g/mol; Hg: 200.59 g/mol). The HBV_Se_ was then calculated to determine the toxicological balance in the edible tissue using Equation (6), as defined by Bautista et al. [39].

HBVSe = ([Se] − [Hg])/[Se] × ([Se] + [Hg])(6)

where

[Se] and [Hg] represent the molar concentrations of selenium and mercury, respectively.

A positive HBV_Se_ value indicates that the Se concentration is sufficient to counteract potential Hg toxicity, signifying that consumption is unlikely to pose a risk. Conversely, negative values suggest a potential Hg-related health risk to consumers due to insufficient Se protection.

### 2.5. Statistical Analysis

All statistical analyses were performed using SPSS for Windows (Version 21.0). Before conducting the parametric analysis, the data were evaluated for normality and homogeneity of variances to ensure they met the necessary assumptions. A Two-way Analysis of Variance (ANOVA) was then employed to determine the effects of sampling station, sex, and their potential interactions on the morphometric characteristics and heavy metal concentrations in shrimp muscle tissues. The explanatory power of the model was assessed using the Adjusted R-squared (Adj. R^2^) value. When significant differences were identified (*p* < 0.05), Duncan’s multiple range test was performed as a post hoc test for multiple comparisons. All results are reported as mean ± standard deviation (SD).

## 3. Results

### 3.1. The Morphometric Characteristics and Meat Yield

In this study, the morphometric characteristics and meat yield of *P. semisulcatus* were evaluated to determine potential sex-based variations across four distinct locations (Bozyazi, Silifke, Karatas, and Iskenderun) along the Northeastern Mediterranean coast. The biometric data, including Whole Weight (WW), Total Length (TL), Meat Weight (MW), and Meat Yield percentage (% MY), are summarized in Table 2.

Statistical analysis (Two-way ANOVA) revealed distinct patterns for growth parameters. Whole Weight (WW) was significantly influenced by both gender (F = 79.80, *p* < 0.001) and sampling station (F = 3.72, *p* < 0.05). Similarly, Meat Weight (MW) showed a strong gender dependency (F = 54.47, *p* < 0.001) and a significant station-gender interaction (F = 4.38, *p* = 0.014). In contrast, Total Length (TL) was significantly affected only by gender (F = 65.02, *p* < 0.001), while spatial variations were not significant (*p* > 0.05). The results demonstrated significant sexual dimorphism in terms of growth parameters. Across all sampled stations, females were significantly larger than males in both body length and weight. For instance, the highest average whole weight was recorded in females from Station 1 (Bozyazi) at 67.53 ± 5.48 g, whereas males from the same station averaged 34.25 ± 2.81 g. Similarly, females from Station 4 (Iskenderun) reached an average weight of 58.00 ± 12.66 g, significantly surpassing the males (33.50 ± 5.35 g). As shown in Table 2, Duncan’s multiple range test indicated significant spatial differences for Whole Weight; Station 1 (Bozyazi) exhibited the highest values (marked with ‘b’), differing significantly from Station 2 (Silifke, marked with ‘a’). Stations 3 and 4 showed intermediate values (marked with ‘ab’), indicating no statistically significant difference from either group.

Regarding the meat yield efficiency (% MY), an inverse trend was partially observed relative to body size. While females provided higher absolute meat weight (MW) due to their larger size, the percentage of meat yield was generally comparable between sexes. Notably, males at Station 1 and Station 4 exhibited the highest meat yield percentages (59.84% and 59.50%, respectively). However, ANOVA results indicated that these differences in Meat Yield percentage were not statistically significant for either gender (F = 2.98, *p* = 0.097) or station (F = 0.57, *p* = 0.643). Consequently, no superscript letters were assigned to the TL, MW, and % MY columns in Table 2, reflecting the absence of significant station-based variations for these parameters. This suggests that while females accumulate more total biomass, the proportion of muscle tissue relative to total body weight remains statistically stable across sexes and locations. Furthermore, during the macroscopic (visual) inspection and morphometric measurements of the collected specimens, no morphological malformations or structural abnormalities were observed in the individuals from any of the sampling stations. This indicates that the current environmental conditions and bioaccumulation levels have not induced visible phenotypic changes or physical deformities in the local shrimp populations.

### 3.2. General Overview of Metal(loid) Concentrations

The mean concentrations of 12 analyzed metals (As, Cd, Co, Cr, Cu, Fe, Hg, Mn, Ni, Pb, Se, and Zn) in the muscle tissues of *P. semisulcatus* across four sampling stations (Bozyazi, Silifke, Karatas, and Iskenderun) and both sexes (female and male) are summarized in Table 3.

While Cobalt (Co) levels were consistently below the detection limits (ND) in all samples, the general accumulation hierarchy for the detected elements was found to be Zn > Fe > Cu > As > Cr > Pb > Se > Mn > Ni > Cd > Hg.

The highest total metal burden was observed in a specimen from Bozyazi (Station 1), whereas the lowest concentrations were generally exhibited by specimens from Karatas (Station 3). Notably, the concentration of highly toxic elements like Hg and Cd remained within a relatively low range compared to essential elements, yet showed significant station-specific spikes, particularly at Iskenderun (Station 4).

### 3.3. Metal(loid) Accumulation in the Muscle Tissue of P. semisulcatus and Spatial Variations

The Two-way ANOVA revealed distinct patterns of bioaccumulation based on station, sex, and their interaction for each metal. The detailed concentrations are presented in Table 3.

#### 3.3.1. Arsenic (As) Levels

**Figure 2:**
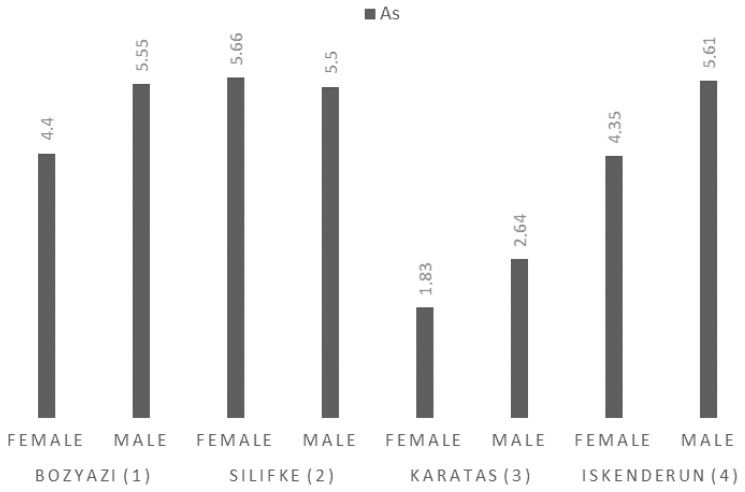
Mean As concentrations (mg/kg wet weight) in the muscle tissue of *P. semisulcatus* by sampling station and sex.

The sampling station had a highly significant effect on As levels (F = 42.090, *p* < 0.001), while sex also exerted a significant influence (F = 10.965, *p* = 0.004). No significant interaction was observed between these two factors (*p* = 0.162), with an adjusted R^2^ of 0.855. The mean As concentrations in the muscle tissue of *P. semisulcatus* ranged from 1.83 ± 0.22 mg/kg (Karatas, Female) to 5.66 ± 0.67 mg/kg (Silifke, Female). The highest values were consistently measured in the Silifke (Station 2) and Iskenderun (Stations 4), with males in Iskenderun reaching 5.61 ± 0.73 mg/kg (Figure 2). Specifically, the lowest measured value in Karatas was approximately 18 times higher than the regulated limit.

#### 3.3.2. Cadmium (Cd) and Chromium (Cr) Levels

Cadmium (Cd) concentrations in the muscle tissue of *P. semisulcatus* were significantly influenced by both sampling station (F = 81.0, *p* < 0.001) and sex (F = 30.76, *p* < 0.001). A clear trend of increasing accumulation was observed toward Iskenderun, where the highest Cd level was measured in males (0.041 ± 0.005 mg/kg). Across all sampling stations and sexes, Cd concentrations ranged from 0.014 ± 0.001 mg/kg to 0.041 ± 0.005 mg/kg.

**Figure 3:**
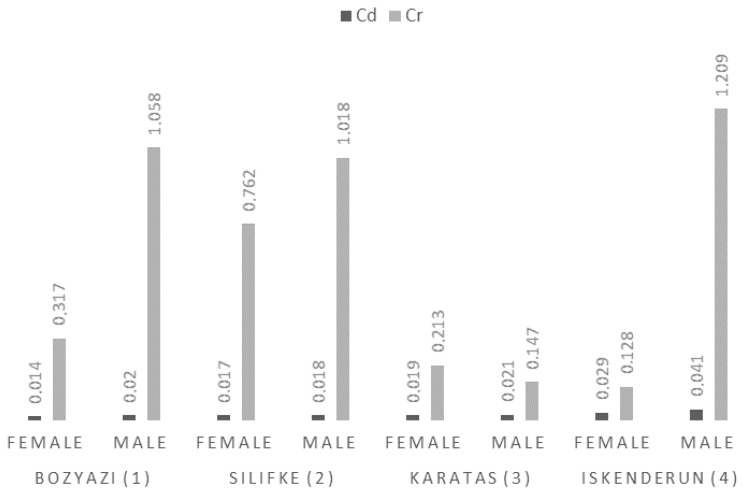
Mean Cd and chromium Cr concentrations (mg/kg wet weight) in the muscle tissue of *P. semisulcatus* by sampling station and sex.

For Cr, a pronounced sex effect was observed (F = 342.27, *p* < 0.001), with the model showing an excellent fit (Adj. R^2^ = 0.978). Cr levels peaked markedly in Iskenderun males (1.209 ± 0.15 mg/kg), which was substantially higher than the levels observed in females at the same station (0.128 ± 0.01 mg/kg) (Figure 3). This significant difference indicates a strong sex-specific accumulation for Cr in certain environments. While the highest Cr value was recorded in Iskenderun, the lowest values were measured in Karatas males (0.147 ± 0.02 mg/kg) and Iskenderun females (0.128 ± 0.01 mg/kg).

#### 3.3.3. Copper (Cu) Levels

The Two-way ANOVA results for Copper (Cu) concentrations indicated that both the sampling station (F = 67.643, *p* < 0.001) and sex (F = 13.983, *p* = 0.002) had statistically significant main effects. Furthermore, a significant interaction between station and sex was observed (F = 5.093, *p* = 0.012), suggesting that the influence of sex on Cu bioaccumulation varies across different geographic locations. The adjusted R^2^ value of 0.907 confirms that the model explains approximately 90.7% of the total variance in Cu concentrations.

**Figure 4:**
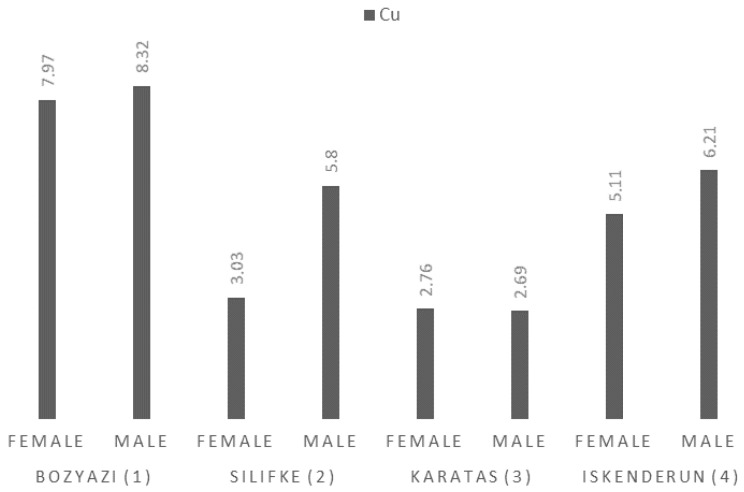
Mean Cu concentrations (mg/kg wet weight) in the muscle tissue of *P. semisulcatus* by sampling station and sex.

Cu concentrations in the muscle tissue of *P. semisulcatus* ranged from 2.69 ± 0.30 mg/kg (Karatas, Male) to 8.32 ± 1.00 mg/kg (Bozyazi, Male) (Figure 4). Notably, Cu concentrations were distinctively higher in Bozyazi (7.97–8.32 mg/kg) compared to other stations, which was confirmed by the distinct statistical groupings.

#### 3.3.4. Iron (Fe) and Mercury (Hg) Levels

**Figure 5:**
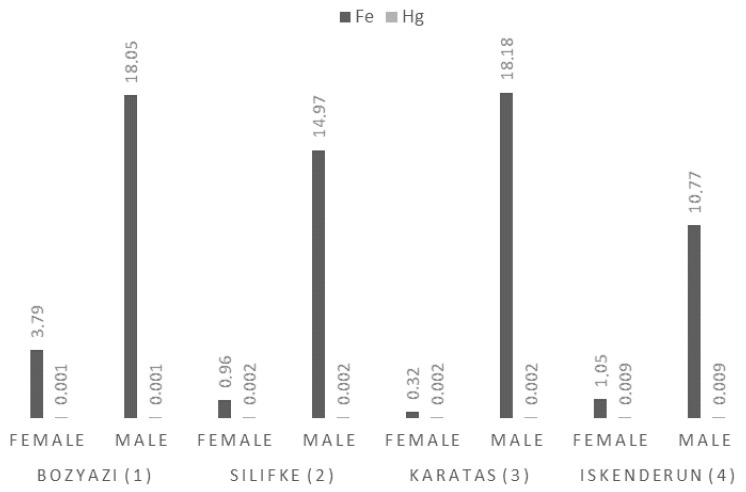
Mean Fe and Hg concentrations (mg/kg wet weight) in the muscle tissue of *P. semisulcatus* by sampling station and sex.

Fe accumulation in the muscle tissue of *P. semisulcatus* was predominantly influenced by sex (F = 654.79, *p* < 0.001), with males generally exhibiting significantly higher levels than females. The highest Fe concentrations were recorded in Karatas males (18.18 ± 2.32 mg/kg), followed by Bozyazi males (18.05 ± 1.89 mg/kg). In contrast, the lowest Fe levels were measured in Karatas females (0.32 ± 0.03 mg/kg) (Figure 5).

Hg levels were primarily determined by the sampling station (F = 3452.63, *p* < 0.001), showing no variation based on sex (*p* = 1.000). This suggests that Hg bioaccumulation in this species is independent of biological factors, as evidenced by the high model fit (Adj. R^2^ = 0.998). Hg concentrations in Iskenderun (0.009 mg/kg) were approximately 9 times higher than those measured in Bozyazi (0.001 mg/kg).

#### 3.3.5. Lead (Pb) and Zinc (Zn) Levels

**Figure 6:**
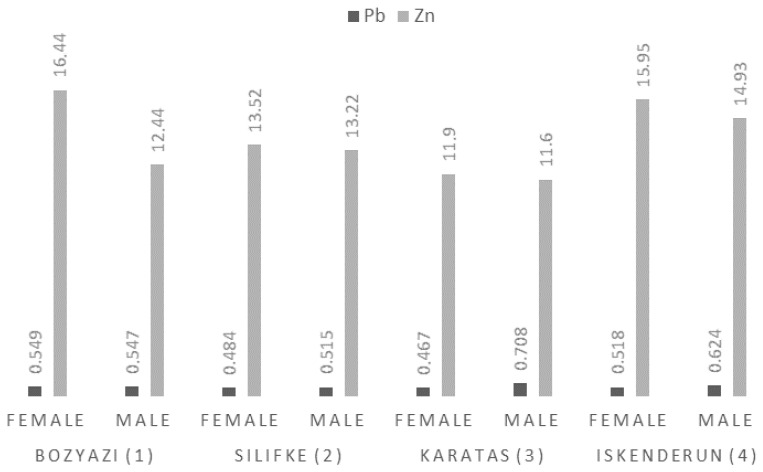
Mean Pb and Zn concentrations (mg/kg; wet weight) in the muscle tissue of *P. semisulcatus* by sampling station and sex.

Lead (Pb) was unique among the analyzed elements as the station effect was non-significant (*p* = 0.141), which is reflected by the lack of differentiating superscripts in Table 3. However, sex exerted a significant influence (*p* = 0.003), with concentrations ranging from 0.467 ± 0.06 mg/kg to 0.708 ± 0.09 mg/kg(Figure 6).

Regarding Zinc (Zn), the station effect was significant (*p* = 0.009), while the influence of sex remained borderline non-significant (*p* = 0.055). The highest Zn levels were recorded in Bozyazi females (16.44 ±1.99 mg/kg) and Iskenderun females (15.95 ± 2.05 mg/kg) (Figure 6).

#### 3.3.6. Manganese (Mn), Nickel (Ni), and Selenium (Se)

**Figure 7:**
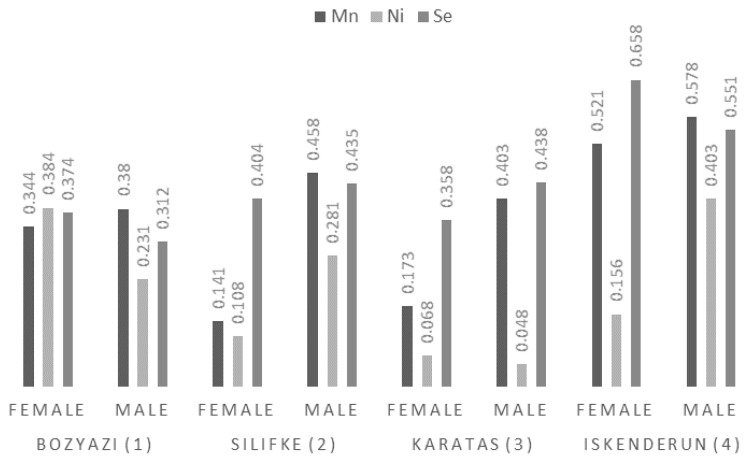
Mean Mn, Ni, and Se concentrations (mg/kg wet weight) in the muscle tissue of *P. semisulcatus* by sampling station and sex.

The statistical analysis for Mn and Ni revealed highly significant interactions between station and sex (*p* < 0.001). Mn levels peaked in specimens collected from Iskenderun (0.521–0.578 mg/kg), while the lowest concentrations were observed in Silifke females (0.141 ± 0.01 mg/kg). Ni concentrations followed a similar pattern of geographic and biological variability, with the highest values recorded in Iskenderun males (0.403 ± 0.05 mg/kg) and Bozyazi females (0.384 ± 0.04 mg/kg) (Figure 7).

Se levels were found to be primarily station-dependent (*p* < 0.001), whereas sex had no overall significant effect (*p* = 0.382). Despite the non-significant main effect of sex, a significant interaction between station and sex was observed (*p* = 0.003). Se concentrations exhibited a distinct geographic gradient, with maximum values measured in Iskenderun (0.551–0.658 mg/kg) and minimum values in Bozyazi (0.312–0.374 mg/kg).

### 3.4. Health Risk Assessment

#### 3.4.1. Estimated Weekly Intake (EWI) of Metals for Green Tiger Shrimp (*P. semisulcatus*)

The potential health risks associated with the consumption of *P. semisulcatus* were evaluated by calculating the Estimated Weekly Intake (EWI) for eleven metal(loid)s. These calculated values were compared against the Provisional Tolerable Weekly Intake (PTWI) guidelines established by the Joint FAO/WHO Expert Committee on Food Additives [40,41] to assess consumer safety (Table 4).

The assessment revealed that the EWI values for all analyzed elements, across all four sampling stations and both sexes, were significantly below the established PTWI thresholds. This indicates that the consumption of *P. semisulcatus* from the Northeastern Mediterranean does not pose an appreciable health risk to adult consumers.

The risk assessment for As was conducted based on the toxicologically relevant inorganic fraction (assumed as 10% of total As). The highest EWI for inorganic As was recorded in females from Silifke (Station 2) at 2.32 × 10^−3^ mg/kg bw/week. This value represents only 15.4% of the provisional safety limit (0.015 mg/kg bw/week), confirming that arsenic exposure through shrimp consumption is well within safe limits.

The EWI values for Cd exhibited spatial variability, with the highest intake observed in males from the Iskenderun region (Station 4) at 1.68 × 10^−4^ mg/kg bw/week. Despite this localized elevation, the value remains substantially below the PTWI of 0.007 mg/kg bw/week, constituting less than 2.5% of the permissible weekly load.

Hg intake was notably low across all stations. However, a distinct spatial pattern was observed; specimens from Iskenderun (Station 4) exhibited the highest Hg EWI (3.69 × 10^−5^ mg/kg bw/week). Nevertheless, these values are orders of magnitude below the PTWI for total mercury (0.004 mg/kg bw/week), posing no toxicological concern.

The EWI for Pb ranged from 1.92 × 10^−3^ to 2.90 × 10^−3^ mg/kg bw/week. Statistical analysis indicated that, unlike other metals, the variation in Pb intake among stations was not statistically significant (*p* > 0.05). All calculated Pb intakes remained well below the PTWI of 0.025 mg/kg bw/week, reaching approximately 11% of the limit at maximum exposure.

Zn was the dominant trace element in terms of intake, peaking at 6.74 × 10^−2^ mg/kg bw/week in females from Bozyazi (Station 1), which is negligible compared to the PTWI of 7.0 mg/kg bw/week. Similarly, Fe intake peaked at levels far below the safety guideline of 5.6 mg/kg bw/week. Likewise, the EWI values for Cu, Cr, Mn, Ni, and Se were also found to be negligible, constituting only a small fraction of their respective PTWI limits and confirming their safety for consumption.

In summary, while spatial factors—particularly the industrial influence of Iskenderun—appear to modulate the intake levels of specific metals like Hg and Cd, the overall EWI profile for *P. semisulcatus* in the Northeastern Mediterranean indicates that this species is entirely safe for human consumption according to international standards. Nevertheless, assessing elements in isolation may not fully capture the actual health burden, as consumers ingest these metals simultaneously. To provide a more realistic evaluation of the combined impact of this metal mixture, the following sections extend the analysis to cumulative non-carcinogenic and carcinogenic risks through THQ and CR indices.

#### 3.4.2. Target Hazard Quotient (THQ) and Total THQ (∑THQ)

The non-carcinogenic health risks associated with the consumption of *P. semisulcatus* were evaluated using the Target Hazard Quotient (THQ) and Total THQ (∑THQ) indices. The calculated values for eleven metal(loid)s across four sampling stations are presented in Table 5.

The assessment revealed a distinct spatial and elemental variation in potential health risks. The Total THQ (∑THQ) values ranged from a minimum of 0.538 in females from Karatas (Station 3) to a maximum of 1.626 in males from Iskenderun (Station 4).

While the cumulative risk in Karatas (Station 3) remained consistently below the safety threshold of 1 for both sexes (∑THQ < 1), indicating no appreciable non-carcinogenic risk, exceedances were observed in the other stations. Specifically, ∑THQ values surpassed 1 in Bozyazi (Station 1), Silifke (Station 2), and Iskenderun (Station 4), driven primarily by two elements: As and Cr.

Even after adjusting for the toxicologically relevant inorganic fraction (10%), As emerged as the dominant risk driver across most stations. Individual THQ values for As exceeded or approached the threshold of 1 in several cases, peaking at 1.105 in females from Silifke (Station 2). This suggests that inorganic arsenic is the primary contributor to the non-carcinogenic risk profile in this species.

Regarding Cr, a notable finding was observed. While CRs were generally low, males from Bozyazi (Station 1) and Iskenderun (Station 4) exhibited elevated THQ for Cr (0.206 and 0.236, respectively). This localized spike in Iskenderun males contributed to elevating the cumulative risk (∑THQ = 1.626) for this specific group.

In contrast, the contributions of other toxic metals, such as Cd, Pb, and Hg, were negligible. For instance, the highest THQ for Pb was 0.012, and for Hg, it was 0.053, both remaining well below the level of concern.

In summary, although the EWI analysis indicated safety based on weekly intake limits, the THQ assessment highlights a potential non-carcinogenic risk arising specifically from As (region-wide) and Cr (localized to specific stations). This discrepancy underscores the importance of a multi-index risk assessment, particularly for populations with high seafood consumption rates.

#### 3.4.3. Target Carcinogenic Risk (CR)

The carcinogenic risk (CR) associated with exposure to As, Cd, Cr, Ni, and Pb through the consumption of *P. semisulcatus* muscle tissue was evaluated, and the results are presented in Table 6.

The calculated CR values were evaluated based on the target risk range of 10^−6^ to 10^−4^, which is established as a benchmark for human health risk assessment by the US EPA [42] and supported by recent literature [35]. The assessment revealed significant spatial variability in cancer risk levels. Among the analyzed elements, As, Ni, and Cr emerged as the primary contributors to the total carcinogenic burden. Even after correcting for the toxicologically relevant inorganic fraction (10%), As posed a considerable baseline risk across all stations, with CR values consistently ranging from 5.97 × 10^−5^ to 1.85 × 10^−4^.

While As presented a generalized risk, critical exceedances were observed for Cr and Ni, particularly in male specimens. The highest individual risk for Cr was recorded in males from Iskenderun (Station 4) with a CR value of 1.31 × 10^−4^, which surpasses the upper tolerance limit of 10^−4^. Similarly, Ni also exhibited elevated risk values, peaking at 1.49 × 10^−4^ in the same group. This finding aligns with the elevated THQ values observed for these metals, pointing to a specific source of pollution likely associated with industrial activities in the Iskenderun Bay. In contrast, the carcinogenic risks associated with other metals were substantially lower; Cd values were negligible (in the 10^−8^ range), while Pb values contributed less significantly to the cumulative risk profile.

Consequently, the Total Carcinogenic Risk (∑CR) values revealed a concerning trend. While the cumulative risk in Karatas (Station 3) was the lowest among the stations (peaking at 1.79 × 10^−4^), the total risk for males in Iskenderun (Station 4) reached 5.15 × 10^−4^. This cumulative value exceeds the upper acceptable risk threshold of 10^−4^ by a factor of approximately five, suggesting that lifetime consumption of *P. semisulcatus* from this heavily industrialized region may pose a potential carcinogenic health risk, predominantly driven by the cumulative effect of As, Ni, and Cr exposure.

#### 3.4.4. Se:Hg Molar Ratios and Selenium Health Benefit Value (HBV_Se_)

The protective role of Selenium against Mercury toxicity was evaluated using the Se:Hg molar ratio and the Selenium Health Benefit Value (HBV_Se_). The results are summarized in Table 7.

Consistent with the low mercury concentrations detected in the tissues, the calculated Se:Hg molar ratios were remarkably high across all sampling stations, ranging from 155.53 in males from Iskenderun (Station 4) to 950.11 in females from Bozyazi (Station 1). These values are orders of magnitude above the critical threshold of 1:1, confirming that selenium is present in large molar excess relative to mercury.

Furthermore, the HBV_Se_ values were consistently positive for all analyzed samples, ranging from 0.312 to 0.658. Since positive HBV_Se_ values indicate a selenium surplus that can effectively sequester mercury and prevent its toxic effects, these findings suggest that the consumption of *P. semisulcatus* from the Northeastern Mediterranean poses no risk of mercury toxicity; on the contrary, it provides a net nutritional benefit in terms of selenium supply.

## 4. Discussion

This study investigated the metal accumulation in the muscle tissue of the commercially important shrimp *P. semisulcatus* from the Northeastern Mediterranean, considering both geographical location and sex. The findings offer significant implications for both regional pollution dynamics and food safety.

### 4.1. Sexual Dimorphism and Metabolic Differences in Metal Accumulation

The morphometric analysis confirmed the expected sexual dimorphism in *P. semisulcatus*, with females consistently exhibiting greater Whole Weight (WW) and Total Length (TL) compared to males (Table 2). This size difference is consistent with recent observations by Mohamed et al. [43]. Generally, aquatic bioaccumulation models suggest a positive correlation between body size and metal burden. However, our data revealed an inverse trend. Despite having significantly lower biomass, males—especially in the locations of Bozyazi (Station 1) and Iskenderun (Station 4)—accumulated much higher concentrations of heavy metals like Fe, Cr, and As. For instance, Fe levels in males at Station 1 were nearly five times higher than in females (18.05 vs. 3.79 mg/kg). This inverse relationship aligns with the metabolic principles described by Balzani et al. [44]. Although their study focused on age-size relationships, they highlighted that smaller individuals often exhibit higher metal concentrations due to faster metabolic rates and higher relative ingestion rates. In our case, the smaller males likely possess a higher metabolic turnover per unit of body mass compared to the larger females, leading to a more rapid uptake of contaminants from the sediment. Therefore, the elevated metal levels in males appear to be driven by these size-dependent metabolic differences rather than simple time-dependent accumulation. Future seasonal studies would also benefit from tracking these sex-specific differences across different reproductive and molt cycle stages to fully elucidate the temporal dynamics of metal accumulation.

A notable finding of this study is the evidence of a localized, sex-specific accumulation of Cr. At Station 4 (Iskenderun), male individuals exhibited a Cr concentration of 1.209 mg/kg, which was approximately 10-fold higher than the 0.128 mg/kg observed in females from the same station (Table 3). This finding offers critical insight when compared to regional literature. For instance, Yipel and Tekeli [45] reported Cr levels as below the limit of detection (<LOD) for the same species in Iskenderun Bay. Our results suggest that previous studies relying on pooled samples (mixing sexes) or broader sampling grids may have diluted and effectively masked these specific “hotspots” of contamination. Furthermore, our recorded value for males (1.209 mg/kg) is more than double the maximum concentration (0.56 mg/kg) reported by Kaymacı and Altun [2] for *P. semisulcatus* in the region. This observation strongly indicates a localized source of Cr input, likely linked to the iron-steel and heavy industries operating in the bay. While our study measures bioaccumulation in tissue, not sediment, the magnitude of this finding reflects a habitat under severe anthropogenic stress. The pollution levels in the bay’s sediment are likely to be classified as ‘heavily to extremely polluted’ according to sediment quality guidelines like those proposed by Förstner and Salomons [46]. Therefore, the high Cr levels in shrimp tissue serve as a direct biological indicator of this severe environmental contamination. The pronounced sex-specific difference also suggests that male *P. semisulcatus* may be more vulnerable to Cr accumulation, potentially due to sex-related differences in metabolic rates, detoxification capacity, or feeding ecology. Crucially, this accumulation pattern aligns with our health risk assessment, where the highest Carcinogenic Risk (CR) for Cr was also identified in this specific group (1.31 × 10^−4^). Ultimately, this “Iskenderun Effect” validates the earlier observations of Yılmaz and Yılmaz [21], demonstrating that sex-based sampling is essential for detecting acute bioaccumulation patterns that general monitoring protocols might otherwise fail to capture. An important ecological consideration is the potential for coastal migration and population mixing between adjacent sampling sites. While this possibility exists, the sheer magnitude of the “Iskenderun Effect”—an order-of-magnitude increase in Cr levels compared to all other sites, including the nearby Karatas—strongly indicates that the influence of the immediate, highly contaminated habitat is powerful enough to override any baseline signature from migrating individuals. The detection of such a sharp, localized signal despite potential population flux further underscores the severity of the anthropogenic pressure within Iskenderun Bay.

### 4.2. Geographical Variations, Pollution Hotspots, and Habitat Influence

To contextualize these geographical variations, it is crucial to reiterate the role of Bozyazi (Station 1) as the designated reference site for this study. While no coastal area in the Mediterranean can be considered entirely free from anthropogenic influence, Bozyazi is characterized by a notable absence of the direct, heavy industrial activities and dense maritime traffic that define stations like Iskenderun Bay. Therefore, the metal concentrations observed in the Bozyazi population serve as a regional baseline, allowing us to interpret the elevated levels at other stations not merely as presence, but as a significant deviation from this reference condition, strongly pointing towards localized pollution sources.

Comparing our findings with historical data allows for a comprehensive assessment of pollution trends across the Northeastern Mediterranean. Our study encompasses a wide spatial gradient, ranging from the relatively unpolluted waters of Bozyazi (Station 1) to the heavily industrialized Iskenderun Bay (Station 4). This spatial heterogeneity facilitates a detailed evaluation of how heavy metal accumulation in *P. semisulcatus* varies in response to anthropogenic pressure, sex-specific physiology, and habitat characteristics. A critical evaluation of our data reveals distinct spatial patterns when juxtaposed with recent regional studies, such as Kosker [10] in Mersin Bay and Yipel and Tekeli [45] in Iskenderun Bay.

Arsenic (As) was identified as a widespread contaminant across the entire Northeastern Mediterranean coast. We detected As levels ranging from 1.83 to 5.66 mg/kg (Table 3), with notably high concentrations even in the non-industrialized Station 1 (Bozyazi). This observation is crucial because statistical analysis showed no significant difference between the reference site and the industrial zone. This finding is strongly corroborated by the recent study of Yipel and Tekeli [45], who reported a mean As concentration of 4.45 mg/kg in *P. semisulcatus* from Iskenderun Bay. The consistency between our findings, Yipel and Tekeli [45], and earlier reports by Kaya and Turkoglu [3] suggests that elevated As is not merely a localized industrial issue. Instead, it likely stems from a widespread combination of agricultural runoff and the region’s specific geochemical background. This conclusion is strongly supported by Micheline et al. [47], who reported even higher As concentrations (7.36–10.48 mg/kg ww) in the lessepsian shrimp *Marsupenaeus japonicus* along the neighboring Lebanese coast. The presence of such high levels across the entire Levantine Basin confirms that elevated As is a characteristic regional feature rather than a site-specific industrial anomaly. This is particularly evident when our results are contrasted with the significantly lower As concentration of 0.249 ± 0.116 mg/kg reported by Yarsan et al. [48] in the same species from the Gulf of Antalya, suggesting a substantial regional difference or a temporal increase in contamination over the past decade.

A significant divergence appears regarding Lead (Pb). While Yipel and Tekeli [45] reported Pb levels as non-detectable (<LOD) in their samples, our study detected Pb concentrations ranging between 0.467 and 0.708 mg/kg across all stations. As highlighted by Abd-Elghany et al. [49], Pb is a non-essential, harmful metal that enters aquatic environments primarily through anthropogenic activities. Therefore, its presence in our samples serves as a direct indicator of human-induced pollution, regardless of concentration magnitude. However, contrary to the expectation of localized industrial pollution, our statistical analysis indicated no significant difference between stations (*p* > 0.05). This finding becomes particularly interesting when positioned within the historical and regional context. Our Pb values are lower than the 0.80–1.04 mg/kg range reported by Kaymacı and Altun [2] for the same species, but distinctly higher than the non-detectable levels of Yipel and Tekeli [45]. Furthermore, our results stand in contrast to the findings of Micheline et al. [47], who reported extremely low Pb levels (0.006–0.007 mg/kg ww) for shrimps on the Lebanese coast. The fact that our values are notably higher than those in the southern Levantine Basin suggests that the Northeastern sector—particularly the Mersin-Iskenderun coastline—is subject to a significantly higher ubiquitous lead burden, likely driven by intense maritime traffic and atmospheric deposition characteristic of this heavy industrial zone. This underscores the critical value of our data in revealing risks that are widespread across the region, often overlooked in generalized monitoring.

The accumulation patterns of essential metals, particularly Zn, further emphasize the independence of physiological accumulation from industrial pollution loads. Contrary to expectations, the highest Zn concentrations were not found in the industrial Station 4, but in Station 1 (Bozyazi), reaching 16.44 mg/kg (Table 3). This supports the hypothesis that Zn accumulation is driven primarily by the metabolic requirements of *P. semisulcatus* rather than environmental contamination levels. This observation is further contextualized by the work of Kaymacı and Altun [2], who reported lower Zn concentrations (8.57–11.69 mg/kg) in their study. The fact that our highest values exceed even those previously reported reinforces that these levels are likely governed by biological demand rather than simple environmental availability. This finding aligns with Yılmaz and Yılmaz [21], reinforcing the concept that essential metal levels are regulated homeostatically by the organism.

The influence of the benthic coastal habitat becomes evident when evaluating the entire study area. Our results for terrigenous metals like Fe showed significant variability, with peaks observed in Station 3 (Karatas) and Station 1 (Bozyazi) (up to 18.18 mg/kg). As stated by Abbaspour et al. [50], Fe is an abundant element on earth and biologically essential. Therefore, Fe accumulation in *P. semisulcatus* tissues should be interpreted primarily as a natural physiological baseline. However, the wide range of Fe concentrations in our study (0.32–18.18 mg/kg) contrasts with the more constrained range (5.40–9.79 mg/kg) found by Kaymacı and Altun [2], suggesting that our sampling stations capture a greater degree of habitat-driven variability. The habitat factor remains critical. Comparing our coastal values with deep-water shrimp species studied by Olgunoglu [20] reveals a clear pattern: coastal species are exposed to significantly higher loads of lithogenic metals (Fe) due to their burrowing behavior in sediment-rich waters. While Olgunoglu [20] found non-detectable levels of toxic metals in deep-sea shrimps, our consistent detection of Pb and Cd confirms that the coastal shelf zone acts as a primary sink for pollutants, exposing benthic organisms to higher risks than their deep-sea counterparts. Regarding the remaining analyzed elements (such as Ni, Cu, Mn, and Hg), the observed concentrations were consistent with recent reports for *P. semisulcatus* in the Northeastern Mediterranean. Unlike the distinct spikes observed for Pb, As, and Cr, these metals did not exhibit extreme spatial or sex-specific fluctuations. The levels of essential metals (Cu, Mn) aligned with the physiological ranges reported in similar studies, while toxic metals (Ni, Hg) generally remained within or below the ranges reported in previous regional surveys [10,45]. For instance, our Hg levels (0.001–0.009 mg/kg) were even lower than the already safe levels (0.03 mg/kg ww) reported by Micheline et al. [47] for *M. japonicus* in Lebanon, indicating that mercury bioaccumulation remains a minimal concern for shrimp species in this region. A particularly noteworthy finding emerges when comparing our data with the seasonal study of Kaymacı and Altun [2]. While our Cu values (2.69–8.32 mg/kg) are in strong agreement with their reported range (5.86–8.54 mg/kg), a sharp divergence appears regarding toxic metals. Our measured concentrations for Ni (0.048–0.403 mg/kg) and especially Cd (0.014–0.041 mg/kg) are substantially lower than the ranges they reported (Ni: 1.19–1.97 mg/kg; Cd: 0.10–0.56 mg/kg). Interestingly, regarding Cd, our results occupy an intermediate position in the Levantine Basin. While our Cd levels are distinctly lower than the historical local data of Kaymacı and Altun [2], they appear slightly elevated compared to the ultra-low levels reported by Micheline et al. [47] for the southern Levantine coast (0.002–0.010 mg/kg). This suggests that while the Northeastern Mediterranean (Iskenderun region) is subject to a higher anthropogenic input than the Lebanese coast, the current pollution load is significantly less severe than previously reported, representing a safe regional baseline rather than acute contamination. It should be noted as a limitation of this study that corresponding sediment or detritus samples were not analyzed. While the benthic feeding ecology of *P. semisulcatus* strongly suggests a dietary uptake route for metals, future studies incorporating direct analysis of their food source would be invaluable for definitively confirming these exposure pathways.

### 4.3. Health Risk Implications and Safety Assessment

From a food safety perspective, our findings present a dual picture. On one hand, certain toxic metals pose a potential health concern by exceeding established legal limits. In all sampling stations and for both sexes, the measured total arsenic (tAs) concentrations significantly exceeded the maximum permissible limit of 0.10 mg/kg established for inorganic arsenic [51]. Similarly, several samples exceeded the 0.50 mg/kg maximum level for Lead (Pb) established for crustaceans [52]; specifically, Pb values in both sexes from Bozyazi and Iskenderun, and males from Silifke and Karatas, were found to be above this legal threshold. On the other hand, concentrations of other metals remained well within safety benchmarks. All measured Cd levels remained well below the maximum permissible limit of 0.50 mg/kg [52], and all measured Hg concentrations also remained below the 0.50 mg/kg limit [52]. For essential metals like Cu and Zn, all concentrations were found to be significantly below the benchmarks of 20.0 mg/kg and 50.0 mg/kg, respectively [53]. These initial findings can be further interpreted through detailed risk assessment models.

A critical evaluation reveals a distinct divergence between EWI and THQ indices. Based solely on EWI, the consumption of *P. semisulcatus* appears entirely safe, as all values remained below JECFA [40,41] limits. However, the THQ approach identified potential chronic risks for As. While EWI values for As were safe (<15% of PTWI), THQ values frequently exceeded 1. This contradiction stems from the conservative nature of the THQ model, which assumes total arsenic toxicity. This specific limitation of the THQ model is clearly demonstrated by Micheline et al. [47] in their assessment of the shrimp *Marsupenaeus japonicus* in the Eastern Mediterranean. In their study, the Total THQ reached alarming levels (up to 9.47) primarily due to As. However, when As was excluded from the calculation (TTHQ), the risk index plummeted to negligible levels (~0.019). This stark difference empirically confirms that high THQ values in Mediterranean shrimp are driven almost entirely by the precautionary assumption of inorganic arsenic toxicity, rather than a genuine toxicological threat. Recent literature further supports this, confirming that in crustaceans, toxic inorganic arsenic is metabolically transformed into soluble arsenobetaine, a non-toxic organic form that is rapidly excreted via human urine without causing adverse health effects [54,55]. Therefore, the actual physiological risk is significantly lower than the THQ model suggests. Nevertheless, considering the precautionary principle, habitual daily consumption necessitates balanced seafood consumption advisories.

The localized carcinogenic risk, particularly driven by Cr in Iskenderun Bay, is a notable concern of this study. The Carcinogenic Risk (CR) for Cr in males from Station 4 reached 1.31 × 10^−4^ (Table 6), a value that surpasses the upper acceptable risk threshold of 10^−4^. This specific elevation, while not as extreme as an order of magnitude, is statistically significant and aligns with the heavy industrial profile of Iskenderun Bay, known for its iron-steel and fertilizer plants. This suggests that “high-frequency consumers” in the Iskenderun region may face an increased carcinogenic risk that warrants attention. However, even this elevated risk value should be interpreted with caution regarding chemical speciation. According to Ramos-Filho et al. [56], most of the Cr present in marine animals exists as Cr(III), which is natural and considered a micronutrient essential for blood glucose regulation. This form is considered less toxic because it is not permeable to biological membranes [56]. In contrast, Cr(VI) species are commonly associated with anthropogenic activities and are highly toxic to living organisms [57]. Since certain parts of our study area, such as Iskenderun, are characterized by intense port activities, maritime transportation, filling facilities, and industrial enterprises [25], we adopted a precautionary approach by assuming the presence of toxic Cr(VI) in our risk calculation. However, if the majority of the accumulated chromium is indeed the less toxic Cr(III) form, as suggested by the literature, the actual carcinogenic risk would be significantly lower than the calculated value.

Despite the presence of Hg in the industrial zone (Station 4), the Selenium Health Benefit Value (HBV_Se_) offers a reassuring perspective. Our results showed consistently positive HBV_Se_ values (0.313–0.658) and extremely high Se:Hg molar ratios (up to 950:1). As highlighted by Frías-Espericueta et al. [58] in wild shrimp populations, Selenium acts as an antagonist to Mercury, sequestering it into insoluble mercury-selenide complexes. This protective mechanism is consistent with the “benefit–risk binomial” assessment described by Barone et al. [59], who confirmed that a positive HBV_Se_ index indicates that the Selenium content in seafood is sufficient to alleviate the potential toxic effects of Hg. Since our samples exhibit Se concentrations orders of magnitude higher than Hg, avoiding *P. semisulcatus* due to mercury fears would be counterproductive; the nutritional benefits of Se in these shrimps clearly outweigh the potential risks associated with Hg exposure [58,59].

## 5. Conclusions

This study provides a comprehensive health risk assessment for the commercially important shrimp, *P. semisulcatus*, along a wide spatial gradient of the Northeastern Mediterranean coast, covering Bozyazi, Silifke, Karatas, and Iskenderun. While the species is generally safe for human consumption across the region, our findings demonstrate that seafood safety is a complex interplay of regional industrial load, biological factors, and the specific limitations of risk modeling.

First, this research empirically demonstrates that standard risk models (THQ) significantly overestimate the health risk from As in Mediterranean crustaceans. The high THQ values observed across the stations were identified as a conservative modeling artifact rather than a genuine physiological threat, given that As in shrimp is predominantly in the non-toxic organic form (arsenobetaine). Consequently, public health advisories should move toward more nuanced assessments to avoid issuing unnecessary warnings based solely on total arsenic levels.

Second, a tangible, localized carcinogenic risk was identified in the industrial hotspot of Iskenderun Bay, where the calculated cumulative index (∑CR > 10^−4^) exceeded the priority threshold. Crucially, this risk was specifically detectable in male specimens, which exhibited Cr concentrations approximately 10-fold higher than females. Specifically, the extreme, sex-specific accumulation of Cr in male shrimp from Iskenderun Bay serves as a direct biological indicator of severe industrial pollution, highlighting that sex-based sampling is critical for revealing localized ecotoxicological risks. This “Iskenderun Effect” proves that sex-based sampling is essential to prevent the masking of acute bioaccumulation patterns that general monitoring protocols might otherwise fail to capture. This model-derived risk, based on a precautionary assessment framework, underscores the necessity for targeted monitoring of industrial pollutants in regional hotspots and the adoption of sex-stratified data collection.

Third, our study reveals a widespread, low-level Pb contamination that showed no significant spatial variation across the four stations, with several samples exceeding EU safety thresholds. Conversely, any potential risks from Hg were conclusively mitigated by an overwhelming molar excess of Se at all locations. Consistently positive HBV_Se_ values confirm that the nutritional benefits of selenium in *P. semisulcatus* clearly outweigh any mercury-related concerns, suggesting that avoiding this seafood due to mercury fears would be counterproductive.

In summary, *P. semisulcatus* remains a safe and nutritionally valuable seafood choice across the studied regions of the Northeastern Mediterranean. However, effective risk management requires a transition from generalized assessments to integrated models that account for sex-specific bioaccumulation, regional industrial footprints, and the protective role of Se to provide accurate and transparent guidance to consumers.

## Figures and Tables

**Table 2:** Mean weights (g), total lengths (cm), and meat yield (%) of green tiger shrimp (*P. semisulcatus*).

Station	Gender	WW (g)	TL (cm)	MW	% MY
1	♀	67.53 ± 5.48 ^b^	20.83 ± 0.29 ^a^	36.43 ± 3.07 ^a^	53.95 ± 2.69 ^a^
1	♂	34.25 ± 2.81 ^b^	17.58 ± 1.14 ^a^	20.68 ± 4.30 ^a^	59.84 ± 8.44 ^a^
2	♀	41.60 ± 10.35 ^a^	22.20 ± 1.83 ^a^	24.23 ± 6.10 ^a^	58.18 ± 0.30 ^a^
2	♂	38.12 ± 4.09 ^a^	18.54 ± 0.85 ^a^	22.24 ± 2.73 ^a^	58.27 ± 1.76 ^a^
3	♀	55.75 ± 5.43 ^ab^	20.35 ± 0.67 ^a^	31.60 ± 3.67 ^a^	56.63 ± 2.12 ^a^
3	♂	35.84 ± 3.72 ^ab^	18.34 ± 0.52 ^a^	20.92 ± 1.57 ^a^	58.52 ± 2.28 ^a^
4	♀	58.00 ± 12.66 ^ab^	20.80 ± 1.48 ^a^	34.10 ± 7.49 ^a^	58.78 ± 0.27 ^a^
4	♂	33.50 ± 5.35 ^ab^	17.88 ± 0.98 ^a^	19.92 ± 3.08 ^a^	59.50 ± 1.47 ^a^

1: Bozyazi; 2: Silifke; 3: Karatas; 4: Iskenderun; ♀: Female; ♂: Male; WW: Whole Weight; TL: Total Length; MW: Meat Weight; MY: Meat Yield. Values are expressed as Mean ± Standard Deviation. Different superscript letters (a, b) within the same column indicate statistically significant differences between stations according to Duncan’s multiple range test (*p* < 0.05); values sharing the same superscript letter are not significantly different (*p* > 0.05).

**Table 3:** Mean concentrations (mg/kg wet weight ± SD) of analyzed metal(loid)s in the muscle tissue of *P. semisulcatus* collected from different sampling stations in the Northeastern Mediterranean.

Station	Sex	As	Cd	Co	Cr	Cu	Fe	Hg	Mn	Ni	Pb	Se	Zn
1	♀	4.40 ± 0.45 ^b^	0.014 ± 0.001 ^a^	ND	0.317 ± 0.04 ^c^	7.97 ± 1.04 ^d^	3.79 ± 0.44 ^c^	0.001 ± 0.0001 ^a^	0.344 ± 0.03 ^b^	0.384 ± 0.04 ^c^	0.549 ± 0.06	0.374 ± 0.04 ^a^	16.44 ± 1.99 ^c^
1	♂	5.55 ± 0.70 ^c^	0.020 ± 0.002 ^a^	ND	1.058 ± 0.14 ^c^	8.32 ± 1.00 ^d^	18.05 ± 1.89 ^c^	0.001 ± 0.0001 ^a^	0.380 ± 0.04 ^b^	0.231 ± 0.03 ^c^	0.547 ± 0.06	0.312 ± 0.03 ^a^	12.44 ± 1.55 ^c^
2	♀	5.66 ± 0.67 ^c^	0.017 ± 0.002 ^ab^	ND	0.762 ± 0.08 ^b^	3.03 ± 0.34 ^b^	0.96 ± 0.11 ^b^	0.002 ± 0.0001 ^b^	0.141 ± 0.01 ^a^	0.108 ± 0.01 ^b^	0.484 ± 0.05	0.404 ± 0.04 ^b^	13.52 ± 1.49 ^b^
2	♂	5.50 ± 0.66 ^c^	0.018 ± 0.002 ^ab^	ND	1.018 ± 0.12 ^b^	5.80 ± 0.59 ^b^	14.97 ± 1.83 ^b^	0.002 ± 0.0001 ^b^	0.458 ± 0.05 ^a^	0.281 ± 0.02 ^b^	0.515 ± 0.06	0.435 ± 0.04 ^b^	13.22 ± 1.44 ^b^
3	♀	1.83 ± 0.22 ^a^	0.019 ± 0.00 ^b^	ND	0.213 ± 0.02 ^b^	2.76 ± 0.30 ^a^	0.32 ± 0.03 ^b^	0.002 ± 0.0001 ^b^	0.173 ± 0.02 ^a^	0.068 ± 0.00 ^a^	0.467 ± 0.06	0.358 ± 0.03 ^b^	11.90 ± 1.35 ^a^
3	♂	2.64 ± 0.30 ^a^	0.021 ± 0.001 ^b^	ND	0.147 ± 0.02 ^b^	2.69 ± 0.30 ^a^	18.18 ± 2.32 ^b^	0.002 ± 0.0001 ^b^	0.403 ± 0.03 ^a^	0.048 ± 0.00 ^a^	0.708 ± 0.09	0.438 ± 0.03 ^b^	11.60 ± 1.45 ^a^
4	♀	4.35 ± 0.56 ^b^	0.029 ± 0.002 ^c^	ND	0.128 ± 0.01 ^d^	5.11 ± 0.56 ^c^	1.05 ± 0.11 ^a^	0.009 ± 0.0002 ^c^	0.521 ± 0.04 ^c^	0.156 ± 0.02 ^c^	0.518 ± 0.06	0.658 ± 0.05 ^c^	15.95 ± 2.05 ^c^
4	♂	5.61 ± 0.73 ^c^	0.041 ± 0.005 ^c^	ND	1.209 ± 0.15 ^d^	6.21 ± 0.81 ^c^	10.77 ± 1.33 ^a^	0.009 ± 0.0003 ^c^	0.578 ± 0.05 ^c^	0.403 ± 0.05 ^c^	0.624 ± 0.07	0.551 ± 0.05 ^c^	14.93 ± 1.80 ^c^

1: Bozyazi; 2: Silifke; 3: Karatas; 4: Iskenderun. ♀: Female; ♂: Male; Values are expressed as Mean ± Standard Deviation. Different superscript letters (a, b, c, d) within the same column indicate statistically significant differences between stations according to Duncan’s multiple range test (*p* < 0.05). Stations sharing the same letter are not significantly different from each other. The absence of different letters for Pb indicates that the station effect was non-significant (*p* = 0.141). ND: Not Detected below the detection limits.

**Table 4:** Estimated Weekly Intake (EWI) of metal(loid)s for adult consumers from muscle tissue of *P. semisulcatus* collected from four different sampling stations in the Northeastern Mediterranean. (mg/kg bw/week) compared with Provisional Tolerable Weekly Intake (PTWI—mg/kg bw/week).

Station	Sex	As	Cd	Cr	Cu	Fe	Hg	Mn	Ni	Pb	Se	Zn
1	♀	1.80 × 10^−3 b^	5.74 × 10^−5 a^	1.30 × 10^−3 c^	3.27 × 10^−2 d^	1.55 × 10^−2 c^	4.10 × 10^−6 a^	1.41 × 10^−3 b^	1.57 × 10^−3 c^	2.25 × 10^−3^	1.53 × 10^−3 a^	6.74 × 10^−2 c^
1	♂	2.28 × 10^−3 c^	8.20 × 10^−5 a^	4.33 × 10^−3 c^	3.41 × 10^−2 d^	7.40 × 10^−2 c^	4.10 × 10^−6 a^	1.56 × 10^−3 b^	9.47 × 10^−4 c^	2.24 × 10^−3^	1.28 × 10^−3 a^	5.10 × 10^−2 c^
2	♀	2.32 × 10^−3 c^	6.97 × 10^−5 ab^	3.12 × 10^−3 b^	1.24 × 10^−2 b^	3.94 × 10^−3 b^	8.20 × 10^−6 b^	5.78 × 10^−4 a^	4.43 × 10^−4 b^	1.98 × 10^−3^	1.66 × 10^−3 b^	5.54 × 10^−2 b^
2	♂	2.25 × 10^−3 c^	7.38× 10^−5 ab^	4.17 × 10^−3 b^	2.38 × 10^−2 b^	6.14 × 10^−2 b^	8.20 × 10^−6 b^	1.88 × 10^−3 a^	1.15 × 10^−3 b^	2.11 × 10^−3^	1.78× 10^−3 b^	5.42 × 10^−2 b^
3	♀	7.50 × 10^−4 a^	7.79 × 10^−5 b^	8.73 × 10^−4 b^	1.13 × 10^−2 a^	1.31 × 10^−3 b^	8.20 × 10^−6 b^	7.09 × 10^−4 a^	2.79 × 10^−4 a^	1.92 × 10^−3^	1.47 × 10^−3 b^	4.88 × 10^−2 a^
3	♂	1.08 × 10^−3 a^	8.61 × 10^−5 b^	6.03 × 10^−4 b^	1.10 × 10^−2 a^	7.45 × 10^−2 b^	8.20 × 10^−6 b^	1.65 × 10^−3 a^	1.97 × 10^−4 a^	2.90 × 10^−3^	1.80 × 10^−3 b^	4.76 × 10^−2 a^
4	♀	1.78 × 10^−3 b^	1.19 × 10^−4 c^	5.25 × 10^−4 d^	2.10 × 10^−2 c^	4.31 × 10^−3 a^	3.69 × 10^−5 c^	2.14 × 10^−3 c^	6.40 × 10^−4 c^	2.12 × 10^−3^	2.70 × 10^−3 c^	6.54 × 10^−2 c^
4	♂	2.30 × 10^−3 c^	1.68 × 10^−4 c^	4.95 × 10^−3 d^	2.55 × 10^−2 c^	4.42 × 10^−2 a^	3.69 × 10^−5 c^	2.37 × 10^−3 c^	1.65 × 10^−3 c^	2.56 × 10^−3^	2.26 × 10^−3 c^	6.12 × 10^−2 c^
PTWI (mg/kg\bw/week)		0.015	0.007	0.023	3.5	5.6	0.004	2.5	0.035	0.025	0.066	7

1: Bozyazi; 2: Silifke; 3: Karatas; 4: Iskenderun. ♀: Female; ♂: Male; Different superscript letters (a, b, c, d) within the same column indicate statistically significant differences between stations according to Duncan’s multiple range test (*p* < 0.05). Stations sharing the same letter are not significantly different from each other. The absence of different letters for Pb indicates that the station effect was non-significant (*p* = 0.141). Risk assessment calculations were performed assuming 10% of Total As is Inorganic As.

**Table 5:** Target Hazard Quotient (THQ) and Total THQ (∑THQ) values for metal(loid)s in muscle tissue of *P. semisulcatus* collected from four different sampling stations in the Northeastern Mediterranean.

Station	Sex	As	Cd	Cr	Cu	Fe	Hg	Mn	Ni	Pb	Se	Zn	∑THQ
1	♀	0.859 ^b^	0.008 ^a^	0.062 ^c^	0.117 ^d^	0.003 ^c^	0.006 ^a^	0.001 ^b^	0.011 ^c^	0.009	0.044 ^a^	0.032 ^c^	1.153
1	♂	1.084 ^c^	0.012 ^a^	0.206 ^c^	0.122 ^d^	0.015 ^c^	0.006 ^a^	0.002 ^b^	0.007 ^c^	0.009	0.037 ^a^	0.024 ^c^	1.523
2	♀	1.105 ^c^	0.010 ^ab^	0.149 ^b^	0.044 ^b^	0.001 ^b^	0.012 ^b^	0.001 ^a^	0.003 ^b^	0.008	0.047 ^b^	0.026 ^b^	1.406
2	♂	1.074 ^c^	0.011 ^ab^	0.199 ^b^	0.085 ^b^	0.013 ^b^	0.012 ^b^	0.002 ^a^	0.008 ^b^	0.009	0.051 ^b^	0.026 ^b^	1.488
3	♀	0.357 ^a^	0.011 ^b^	0.042 ^b^	0.040 ^a^	0.0002 ^b^	0.012 ^b^	0.001 ^a^	0.002 ^a^	0.008	0.042 ^b^	0.023 ^a^	0.538
3	♂	0.515 ^a^	0.012 ^b^	0.029 ^b^	0.039 ^a^	0.015 ^b^	0.012 ^b^	0.002 ^a^	0.001 ^a^	0.012	0.051 ^b^	0.023 ^a^	0.712
4	♀	0.849 ^b^	0.017 ^c^	0.025 ^d^	0.075 ^c^	0.001 ^a^	0.053 ^c^	0.002 ^c^	0.005 ^c^	0.009	0.077 ^c^	0.031 ^c^	1.143
4	♂	1.095 ^c^	0.024 ^c^	0.236 ^d^	0.091 ^c^	0.009 ^a^	0.053 ^c^	0.002 ^c^	0.012 ^c^	0.010	0.065 ^c^	0.029 ^c^	1.626

1: Bozyazi; 2: Silifke; 3: Karatas; 4: Iskenderun. ♀: Female; ♂: Male; Different superscript letters (a, b, c, d) within the same column indicate statistically significant differences between stations according to Duncan’s multiple range test (*p* < 0.05). Stations sharing the same letter are not significantly different from each other. The absence of different letters for Pb indicates that the station effect was non-significant (*p* = 0.141). Risk assessment calculations were performed assuming 10% of Total As is Inorganic As.

**Table 6:** Carcinogenic Risk (CR) and Total Carcinogenic Risk (∑CR) values for metal(loid)s in muscle tissue of *P. semisulcatus* collected from four different sampling stations in the Northeastern Mediterranean.

Species	Sex	As	Cd	Cr	Ni	Pb	∑CR
1	♀	1.44 × 10^−4 b^	3.05 × 10^−8 a^	3.45 × 10^−5 c^	1.42 × 10^−4 c^	4.54 × 10^−5^	3.66 × 10^−4^
1	♂	1.81 × 10^−4 c^	4.35 × 10^−8 a^	1.15 × 10^−4 c^	8.54 × 10^−5 c^	4.52 × 10^−5^	4.27 × 10^−4^
2	♀	1.85 × 10^−4 c^	3.70 × 10^−8 ab^	8.29 × 10^−5 b^	3.99 × 10^−5 b^	4.00 × 10^−5^	3.48 × 10^−4^
2	♂	1.79 × 10^−4 c^	3.92 × 10^−8 ab^	1.11 × 10^−4 b^	1.04 × 10^−4 b^	4.26 × 10^−5^	4.37 × 10^−4^
3	♀	5.97 × 10^−5 a^	4.13 × 10^−8 b^	2.32 × 10^−5 b^	2.51 × 10^−5 a^	3.86 × 10^−5^	1.47 × 10^−4^
3	♂	8.62 × 10^−5 a^	4.57 × 10^−8 b^	1.60 × 10^−5 b^	1.78 × 10^−5 a^	5.85 × 10^−5^	1.79 × 10^−4^
4	♀	1.42 × 10^−4 b^	6.31 × 10^−8 c^	1.39 × 10^−5 d^	5.77 × 10^−5 c^	4.28 × 10^−5^	2.57 × 10^−4^
4	♂	1.83 × 10^−4 c^	8.92 × 10^−8 c^	1.31 × 10^−4 d^	1.49 × 10^−4 c^	5.16 × 10^−5^	5.15 × 10^−4^

1: Bozyazi; 2: Silifke; 3: Karatas; 4: Iskenderun. ♀: Female; ♂: Male; Different superscript letters (a, b, c, d) within the same column indicate statistically significant differences between stations according to Duncan’s multiple range test (*p* < 0.05). Stations sharing the same letter are not significantly different from each other. The absence of different letters for Pb indicates that the station effect was non-significant (*p* = 0.141). Risk assessment calculations were performed assuming 10% of Total As is Inorganic As.

**Table 7:** Se:Hg molar ratios and Selenium Health Benefit Values HBV_Se_ in muscle tissue of *P. semisulcatus*.

Station	Sex	[Se]_mol_ (mmol/kg)	[Hg]_mol_ (mmol/kg)	Se:Hg Molar Ratio	HBV_Se_
1	♀	0.00474	4.985 × 10^−6^	950.11	0.374
1	♂	0.00395	4.985 × 10^−6^	792.60	0.312
2	♀	0.00512	9.970 × 10^−6^	513.16	0.404
2	♂	0.00551	9.970 × 10^−6^	552.54	0.435
3	♀	0.00453	9.970 × 10^−6^	454.73	0.358
3	♂	0.00555	9.970 × 10^−6^	556.35	0.438
4	♀	0.00833	4.487 × 10^−5^	185.73	0.658
4	♂	0.00698	4.487 × 10^−5^	155.53	0.551

## Data Availability

The original contributions presented in this study are included in the article. Further inquiries can be directed to the corresponding author.
